# A Systematic Review and Meta-Analysis on the Efficacy of Curcumin/Turmeric for the Prevention and Amelioration of Radiotherapy/Radiochemotherapy Induced Oral Mucositis in Head and Neck Cancer Patients 

**DOI:** 10.31557/APJCP.2021.22.6.1671

**Published:** 2021-06

**Authors:** Sreedevi Dharman, Maragathavalli G, Karpagavalli Shanmugasundaram, Rajesh Kumar Shanmugam

**Affiliations:** 1 *Department of Oral Medicine and Radiology, Saveetha Dental College and Hospitals, Saveetha Institute of Medical and Technical Sciences, Saveetha University, Chennai, Tamil Nadu, India. *; 2 *Department of Oral Medicine and Radiology, Seema Dental College and Hospitals, Rishikesh, India. *; 3 *Department of Pharmacology, Saveetha Dental College, Saveetha Institute of Medical and Technical Sciences, Saveetha University, Chennai, India. *

**Keywords:** Turmeric, curcumin, oral mucositis, radiotherapy, radio-chemotherapy, oral cancer, systematic review

## Abstract

**Background:**

Oral Mucositis(OM) is an acute debilitating dose limiting toxicity of Radiotherapy/Radiochemotherapy(RT/RCT) in management of Head and Neck Cancer (HNC). Curcumin/Turmeric may reduce OM in patients. Aim: Efficacy of Curcumin/Turmeric for preventing and ameliorating the onset and severity of RT/RCT induced OM was analysed in this review.

**Methods:**

A systematic literature search with meta-analysis were performed using Mesh terms in PubMed, Google scholar, Science Direct, Cochrane library and manual searching, articles published from 2010 to April 2021 were included. Clinical trials that studied the efficacy/effects of turmeric / curcumin in management of RT/RCT induced OM in HNC patients were included. Statistical Analysis were done to calculate the pooled Risk ratio at 95%confidence interval with significance at p <0.05.

**Results:**

Nine studies with overall 582 patients with HNC undergoing RT/RCT were included qualitatively. From evidence, orally 1,500-2,000 mg/day of Curcumin/Turmeric, (80mg/day/0.1%mouthwash) of nanocurcumin, topically gel/mouthwash used with increase in frequency prior and the entire course of RT/RCT with long follow ups are beneficial with no serious adverse effects. Meta-analysis of 5 prophylactic trials favoured curcumin/turmeric in reducing the severity of OM (RR 0.48 at 95% CI=0.23,0.99,P=0.05), did not prevent the overall incidence of OM (RR 0.99 at 95%CI=0.95,1.03,P=0.67) but delayed the onset of OM (RR 0.38 at 95% CI=0.18,0.80, P=0.01) during RT/RCT compared to control. Mean mucositis grade (Grade 3) was reduced in curcumin/turmeric with a mean difference of (-0.85 at 95%CI=-1.02,0.67, P<0.00001) over control. Pooling of 2 therapeutic trials favoured Curcumin/Turmeric with significant reduction of pain score at a mean difference of -2.17 at 95%CI =-2.77, -1.58,P<0.00001 over chlorhexidine.

**Conclusion:**

Curcumin/Turmeric are safe, efficacious, well tolerated in preventing the delay in onset and severity of OM, therapeutically ameliorated pain in patients with cancer therapy induced OM. We recommend novel curcumin formulations, high quality randomised controlled trials to further improve its therapeutic effects.

## Introduction

Systemic chemotherapy, radiotherapy or both have become more effective for managing head and neck carcinoma, but they are related with short and long term side-effects (Paiar et al., 2020). Oral mucositis is a common short term side effect that are painful inflammation and ulceration of oral-pharyngeal mucosa (Sonis et al., 2004).

There is functional disruption and integrity of oral mucosa that are acute and noticed as redness to severe ulceration and are infected by fungus such as oral candidiasis (Yuan and Sonis., 2014). High dose chemotherapy induced oral mucositis produce atrophy of the mucosal lining of mouth followed by ulcer formation affecting 100% of patients, radiotherapy induced oral mucositis frequently affecting upto 80% of patients, conventional chemotherapy upto 20-40% (Lalla et al., 2014). Mouth soreness with erythema occurs within 2 weeks of start of radiotherapy, followed by severe epithelial damage within next 2 weeks (Duncan et al., 2005). With concurrent chemoradiotherapy, incidence and severity of OM are still greater (Ver-Llonch et al., 2006). These cancer treatments produced complications causes pain or oral irritation such as difficulty in swallowing and eating, bleeding, reduced nutrition, leading to weight loss, deferral in cancer therapy, extended hospitalisation, expenses and life frightening infections such as septicaemia (Bowen et al., 2019, Murphy et al., 2007).

Based on the severity, OM is classified as tolerable mucositis (grade 1 and 2 mucositis) and intolerable mucositis (grade 3 or more) (Khanal et al., 2010). In HNC undergoing radiotherapy, tolerable mucositis begins in all patients and is manageable, intolerable mucositis requires effective pain management, gastrostomy tube, IV line for nutritional supplementation (Elting et al., 2008). This can also inturn lead to radiotherapy reduction and delay in dose, even ending the planned radiotherapy, thus complicating the underlying cancer therapy (Bensinger et al., 2008).

Presently available treatment are only palliative and for intolerable mucositis not widely accepted treatment is available (Rodriguez-Caballero et al., 2012). Chlorhexidine gluconate is frequently used mouthwash solutions, but literatures doesn’t support much of its use due to its stinging and dehydration, leading to microbial colonisation further increasing the patient’s complication (Cardona et al., 2017). Pain due to OM leads to swallowing difficulty and requires opioid analgesia, which rapidly develop tolerance (Epstein et al., 2019). Honey applied topically inspite of its effectiveness could enhance radiation-related caries (Van den Wyngaert, 2012). Interventions such as basic oral care (Hong et al., 2019), cytokines and growth factors (Logan et al., 2020), cryotherapy (Correa et al., 2020) are suggested by Multinational Association of Supportive Care in Cancer/The International Society of Oral Oncology (MASCC/ISOO) Clinical Practice Guidelines,but none of them were proved to be used as standard treatment.Palifermin (keratinocyte growth factor-1) has been approved by the US Food and Drug Administration but due to its application only for patients on high dose chemotherapy, expensiveness, difficulty to administer, is not considered a better choice (Nooka et al., 2014). Oral zinc sulfate (Tian et al., 2018), Oral glutamine (Anderson and Lalla, 2020),laser therapy (de lima et al., 2020), photobiomodulation therapy (Campos et al., 2020) are recently been tried but definitive therapy has not been established.

Herbal therapy with varied pharmacological benefits with minimal adverse effects are required. Turmeric (Curcuma longa) belongs to Zingiberaceae is a medicinal herb, its active component being curcumin. Curcumin/Turmeric has antioxidant, analgesic, anti-inflammatory, antimicrobial, wound healing agent, has chemosensitizing and radiosensitizing properties (Nagpal and Sood, 2013). Previous studies have already shown that it is effective against proinflammatory cytokines, cyclooxygenase, prostaglandin E. (Maziero et al., 2018) Turmeric stands forefront in wound healing (Mohanty et al., 2017) Studies have proven turmeric/curcumin are effective in potentially malignant disorders like oral submucous fibrosis (Rai et al., 2019), oral lichen planus (Nosratzehi., 2018)

A systematic review (Normando et al., 2019) due to few studies and heterogeneity was present among studies meta-analysis wasn’t performed. A recent meta-analysis reported incidence of severity of oral mucositis(>grade2) alone and subgroup analysis weren’t performed (Zhang et al., 2020). The present systematic review highlights the suggested dosage and appropriate usage, bioavailability aspects of turmeric /curcumin in chemoradiotherapy induced oral mucositis in Head and neck cancer patients. Moreover, three studies have been included two for qualitative synthesis and one published recently for quantitative analysis in our review. Prophylactic studies were analysed into Radiotherapy and Radiochemotherapy, topical and oral mainly to compare the curcumin effects across different subgroups, delay in onset (Grade 1) was also performed to assess the prophylactic effect of curcumin. It was aimed to analyse the existing literature on the efficacy of Curcumin/Turmeric for preventing and mitigating oral mucositis in patients undergoing Chemo-radio therapy for head and neck cancers.

## Materials and Methods

*Methods*

This systematic review followed PRISMA (Preferred Reporting Items for Systematic Review and Meta-analysis) statement.

*Data Source and Search Criteria*

A systematic literature search was done to identify articles describing turmeric/curcumin in OM on chemo/radiotherapy in HNC patients from 2010 till April 2021 were included. Databases searched were PubMed using MeSH terms, science direct, Cochrane library, google scholar. The search methodology applied in PubMed using MeSH terms with following keywords are, ((((((((((((((((((“Curcumin” [MeSH Terms] OR“Curcumin” [All Fields]) OR “curcumin s” [All fields])OR“curcumine”[AllFields])OR“curcumins”[AllFields])OR(((“Curcuma”[MeshTerms]OR“Curcuma”[AllFields]OR“curcumas”[AllFields]OR”curcumae”[AllFields]))“Curcuma” [MeSH Terms] OR(( “Curcuma”[All Fields]) “Curcuma”[MeSH Terms] OR “Curcuma” [All Fields] OR “turmeric”[All Fields]OR “turmeric extract”[Supplementary Concept])AND ((((”Radiotherapy”[MeSH Terms OR “Radiotherapy”[All Fields] OR “radiotherapies” ”[All Fields]OR”Radiotherapy”[MeshSubheading]OR“radiotherapys”[AllFields]))OR”Radiotherapy”[MeSHTerms])OR((((((“chemotherapys”[AllFields]OR“drugtherapy”[MeSHTerms])OR “drug” [All Fields] AND “therapy”[All Fields])) OR“drug therapy” ”[MeSH Subheading]) OR “chemotherapy”[AllFields]))OR”chemotherapy,adjuvant”[MeSHTerms])OR((“Chemotherapy”MeSHTerms]OR“Chemoradiotherapy”[AllFields]OR”chemoradiotherapies”[All Fields])) OR ”Chemoradiotherapy”[MeSH Terms]AND((((“Head and Neck Neoplasms”[MeSH Terms]OR((“head”[All Fields]AND “neck” ”[All Fields] AND “neoplasms” ”[All Fields]))OR “Head and Neck Neoplasms”[All Fields])OR ((“head” ”[All Fields] AND “neck” ”[All Fields]AND “cancer” ”[All Fields] or “head and neck cancer” OR “Head and Neck Neoplasms”[MeSH Terms])AND ((“stomatitis”[MeSH Terms} OR “stomatitis[All Fields]OR (“oral” [All Fields]AND “mucositis” [All Fields])) OR “oral mucositis:[All Fields].

The review search included published and unpublished articles and only those listed in English literature. Manual searches of the articles were also performed. The search results were short listed using preset inclusion and exclusion criteria. The articles were screened on the basis of title and abstract. Full text was then procured for the relevant articles which fulfilled the inclusion criteria. Two reviewers searched and analysed the studies independently. Disagreement between articles was discussed and resolved.

*Eligibility Criteria*

Inclusion Criteria:

Type of Participants: Prevention/Treatment of oral mucositis undergoing radio-chemotherapy in HNC patients.

Types of Interventions: Curcumin/Turmeric

Comparators: Placebo/Other standardised interventions

Type of Studies: Studies with randomised clinical trials, non randomised clinical trials.

Outcome Measures:

Primary Outcome: Incidence of OM and its severity, Delay in onset of OM, Mean Mucositis grade

Secondary Outcome: Mean pain scores, loss of weight

Pain assessment by Visual Analog Scale(VAS), Numeric Rating Scale(NRS), Oral mucositis is assessed by graded scales such as Radiotherapy Oncology Group (RTOG), World Health Association (WHO) scales, Oral Mucositis Assessment Scale (OMAS), National Cancer Institute Common Toxicity Criteria version 2 scale (NCI-CTC v.2), National Cancer Institute Common Terminology Criteria for Adverse Events, version 4.0(NCI CTCAE assessment V.4)

Onset of mucositis-Time of appearance of first sign of mucositis

In RTOG and WHO scales, grade 1 and 2 are tolerable mucositis, degree of severity of mucositis are grade 3 and above which are intolerable type.

Dropouts-Within number of days the patient had to stop the cancer treatment due to mucositis

Weight loss-Weight loss calculated after the completion of therapy.

*Exclusion Criteria*

Animal study, invitro, Editorials/commentaries, surveys, reviews, guidelines were excluded from this systematic review.

*Evaluation of methodological quality*

Nine articles were assessed for their quality using Review manager software using Revman 5.4. Quality assessment of interventional studies include seven domains. Each domain consisted of one question which were answered “low risk” or “high risk” or “unclear risk”. The domains included are Random sequence generation (selection bias), Allocation concealment (selection bias), Blinding of participants and personnel (performance bias), Blinding of outcome assessment (detection bias), Incomplete outcome data (attrition bias), Selective reporting (reporting bias) and Other bias. This data was fed into Review manager software namely in Revman 5.4 to obtain a colour coded chart of risk of bias summary and graph.

*Data Gathering and synthesis*

Data extraction included author, year, study design, Patients gender, age, sample size, groups, grading scales, curcumin formulations, its dosage and directions, results, adverse effects, outcomes. These data were extracted by 2 authors and discussions were done to rule out any differences.

*Statistical Analysis*

Meta-analysis was done using program Review Manager 5.4.1. Pooled effects are calculated when the dichotomous data will be expressed as Relative Risk with 95%CI, continuous data as mean difference with 95%CI. I^2^ method are used to assess the heterogeneity among studies. Heterogeneity was statistically significant if p value was <0.1. I^2^ values of < 25, >25 - <75, >75 suggest low, moderate, high heterogeneity. If heterogeneity was present random effects model was used, if absent fixed effects model are used for overall effects calculation. Funnel Plot was used for the analysis of publication bias 

## Results

*Selection Criteria*

Initial search strategy on PubMed, Google scholar, Science Direct, Cochrane library and gray literature yielded a total of 2,394 articles based on keywords. Number of articles screened by title and abstract were 1,728 after removing duplicates. Records excluded which did not meet the preset criteria based on their abstracts were 1,711. Full text reviewed for eligibility were 21, from which 12 articles were removed for not meeting inclusion criteria. 9 articles totally were finalized for this systematic review based on the entire content of the articles. Only seven of the nine studies with 283 participants were included in this review provided data for meta-analysis on overall incidence of onset and severity of oral mucositis. 

**Figure 1:**
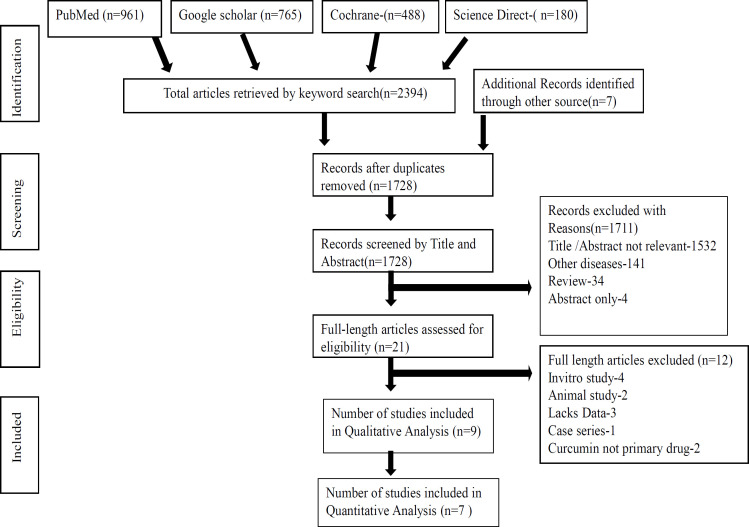
PRISMA Search Flow Chart Showing the Sources and the Final Short Listed Articles Included in This Review

Figure 1 depicts Prisma Flow chart showing the sources and the final short listed articles included in this review.

*Study Design and Duration of included studies*

Seven randomized controlled trials (Rao et al., 2014, Mansourian et al., 2015, Patil et al., 2015, Charanthimath, 2016, Delavarian et al., 2019, Arun et al., 2020, Shah et al., 2021) non randomised controlled trials (Adhvaryu et al., 2018, Saldanha and Almeida, 2014) investigated the efficacy/effects of curcumin in oral mucositis. Evaluation period varied from 5 days to 8 weeks, follow up visits were 5 days (Saldanha and Almeida, 2014), 2 weeks (Charanthimath, 2016), 20 days (Patil et al., 2015), 42 days (Delavarian et al., 2019), 6 weeks (Rao et al., 2014), (Shah et al., 2021), 8 weeks (Mansourian et al., 2015), 2 months (Arun et al., 2020; Adhvaryu et al., 2018).

*Participants Characteristics*

The number of participants who took part in studies varied in size from 20-80 in number. Overall 582 patients with HNC undergoing RT/RCT were included in this systematic review. Male participants were 413, female participants were 129. 1 study didn’t report gender (Charanthimath, 2016). In 6 studies patients underwent radio-chemotherapy (Rao et al., 2014; Patil et al., 2015; Charanthimath, 2016; Arun et al., 2020; Adhvaryu et al., 2018; Saldanha and Almeida., 2014), 3 studies radiotherapy (Mansourian et al., 2015, Delavarian et al.,2019; Shah et al., 2021). Regarding the age, two study didn’t report the age of the participants (Mansourian et al., 2015; Charanthimath, 2016), age range from 26-85 years (Rao et al., 2014), 39-70 years (Patil et al., 2015), 30-90 years (Arun et al., 2020), 10-90 years (Adhvaryu et al., 2018), 31-75 years (Saldanha and Almeida., 2014), 26-96 years (Shah et al., 2021), mean age 62.18+-15.07 (Delavarian et al., 2019). Table 1 shows Characteristics of Included Studies.

*Intervention Characteristics*

9 included studies evaluated the varied drug formulations of Curcumin. 2 clinical trials used Curcumin gel (Mansourian et al., 2015; Charanthimath, 2016). Curcumin mouthrinse 0.004% (Patil et al., 2015), 0.1%Curcumin nanoparticle 10 ml mouthwash (Shah et al., 2021), Turmeric gargle 10 ml (Rao et al., 2014), Turmeric mouthwash (Saldanha and Almeida., 2014), capsule nanomicelle 80 mg (Delavarian et al.,2019), turmeric extract capsule 500 mg (Arun et al.,2020), oral 650 mg Curcumin fortified with 13 mg piperine (Adhvaryu et al., 2018). Interventions were prescribed 2 min 6 times a day(Rao et al.,2014),3 times/day for 7 days (Shah et al., 2021), 3 times a day gel (Mansourian et al., 2015),1:1,1 min thrice daily (Patil et al.,2015), 1 capsule/day orally (Delavarian et al,2016), 1 capsule thrice daily after food from first day of radiation (Arun et al,2020), 3 times a day 3 days prior to start of RT (Adhvaryu et al., 2018), thrice daily for 5 days Saldanha and Almeida., 2014), Patients had to avoid eating or drinking for 15 minutes before the initiation of radiotherapy, they had to cover whole mouth with a thin layer of gel by cotton applicator. Gel was applied for all radiotherapy duration 21 days 3 times a day (Mansourian et al., 2015). Variations were observed in control agents. Chlorhexidine gel (Charanthimath,2016), Placebo topical gel (Mansourian et al., 2015), Chlorhexidine mouthwash (Patil et al.,2015), Povidone Iodine (Rao et al.,2014), Placebo tablets-Lactose (Delavarian et al., 2019), placebo capsules Conventional radio-chemotherapy (Adhvaryu et al., 2018), Saline mouthwash (Saldanha and Almeida., 2014), 0.15% benzydamine mouthwash (Shah et al., 2021).

*Clinical Parameters*

Grading of oral mucositis were assessed by OMAS in 3 studies (Patil et al., 2015), (Charanthimath et al., 2016), (Saldanha and Almeida., 2014), NRS in 2 studies (Patil et al.,2015; Charanthimath, 2016), VAS in 1 study (Mansourian et al., 2015), WHO Mucositis scale in 6 studies (Mansourian et al.,2015; Patil et al., 2015; Charanthimath, 2016; Arun et al., 2020; Adhvaryu et al., 2018; Shah et al., 2021), RTOG in 1 study (Rao et al., 2014), NCI-CTC v.2 in 1 study (Delavarian et al., 2019), NCI CTCAE assessment V.4 (Saldanha and Almeida., 2014)

*Risk of bias*

**Figure 2:**
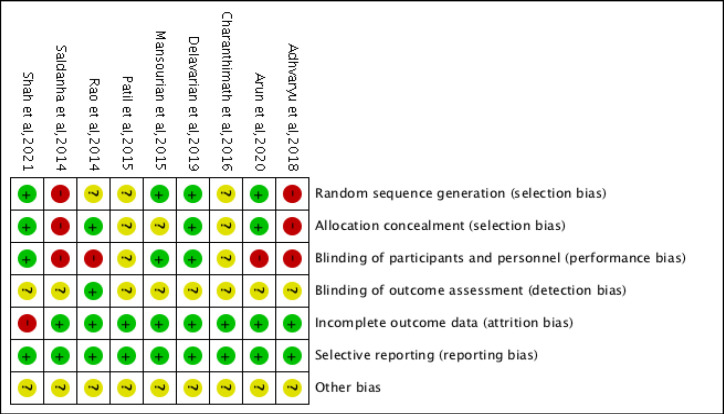
Risk of Bias and Applicability Concerns Summary: review authors judgements about each domain for each included study

**Figure 3:**
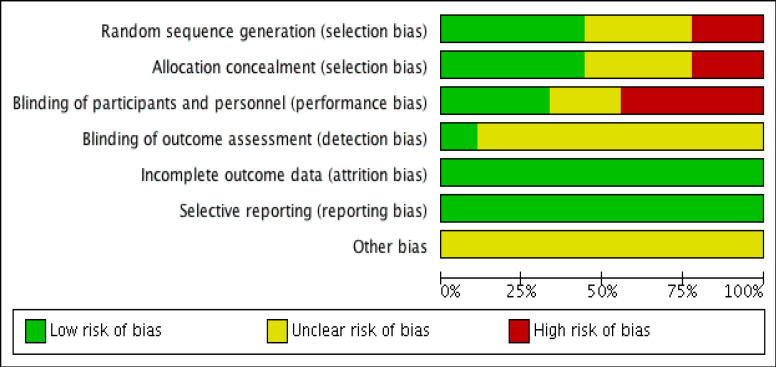
Risk of Bias and Applicability Concerns Graph: review authors' judgements about each domain presented as percentages across included studies

Among 9 studies, according to Cochrane Risk of Bias tool the estimated risk of bias was “low” in 3 studies (Mansourian et al., 2015; Delavarian et al., 2019; Shah et al.,2021), “moderate” in 6 studies (Rao et al.,2014; Patil et al., 2015; Charanthimath, 2016; Arun et al.,2020; Adhvaryu et al., 2018; Saldanha and Almeida., 2014). Only RCTs were assessed for randomization, allocation, blinding, rather non RCTs had either negative or unclear responses. Method used to generate sequence of randomisation was given only by 4 studies (Mansourian et al., 2015; Delavarian et al., 2019), Arun et al., 2018; Shah et al., 2021), balanced block randomization method using computer-generated random number table (Mansourian et al., 2015; Delavarian et al., 2019), 4x4 block randomisation sequence generated (Arun et al., 2020). Allocation concealment were done in 3 studies using opaque envelopes (Rao et al., 2014), identical containers (Delavarian et al., 2019), (Shah et al., 2021) sequentially numbered containers (Arun et al., 2020).1 RCT is triple blinded, parallel arm with intention to treat and Per protocol analysis done (Shah et al., 2021), 2 RCTs (Mansourian et al., 2015), Delavarian et al., 2019) were double blinded, one study had blinding of outcome assessor (Rao et al., 2014). 3 studies had patient withdrawal from study after randomisation (Rao et al., 2014; Delavarian et al., 2019; Shah et al., 2021). Loss to follow up were disclosed and prespecified outcome were reported in studies. All these were considered under low risk of bias. In Shah et al., (2021) there were large loss to follow up, but they were balanced across groups. 2 RCT was single blinded (Rao et al., 2014; Arun et al., 2020) which were considered as high risks. Description not provided due to missing and incomplete information were put under unclear risk. Majority of RCTs had not mentioned flow of participants details, intention to treat analysis, estimated effect size 95%Confidence interval. Figures 2, 3 depicts risk of bias summary and graph for review of authors’ judgements about each domain for the included studies.

*Study Description*

*Effect on Onset of Oral Mucositis*

6 studies (Rao et al., 2014; Delavarian et al., 2019; Arun et al., 2020; Adhvaryu et al., 2018; Shah et al., 2021; Mansourian et al., 2015) assessed the curcumin/turmeric effects on the onset of OM. Delavarian et al., (2019) showed that there was delay in onset of OM in nanocurcumin group compared to control group (P=0.002), only 25% developed grade 1 OM in 2^nd^ week. Arun et al., (2020) reported that patients in turmeric group showed decrease in incidence of OM compared to placebo after 3 weeks of treatment.86.7% of patients had grade 1 OM in turmeric group whereas 71% had grade 2 OM in placebo group. A study by Adhvaryu et al., (2018) observed significant decrease in incidence of OM from 92% to 51% (P ≤ 0.001) among control and curcumin treated group was observed. Onset of tolerable and intolerable mucositis was delayed in the patients using turmeric showed statistically significant difference compared to controls in a study by Suresh Rao et al., (2014). Shah et al.,2021 showed that nine (100%) patients in benzydamine group and six (75%) patients in curcumin group experienced OM . 

*Effect on Degree of severity*

A total of 6 studies (Rao et al.,2014, Mansourian et al.,2015, Delavarian et al.,2019, Arun et al.,2020, Adhvaryu et al.,2018, Shah et al.,2021) assessed the curcumin/turmeric effects on degree of severity. The study by Mansourian et al., (2015) found that there was no grade 3 mucositis, time the symptom started was longer, mean size of oral lesion, erythema, burning sensation in curcumin group was significantly lower than control group(p<0.0001). The study by Rao et al., (2014) reported that turmeric showed statistical significance of (p<0.0001), 14 of 39 patients with intolerable mucositis whereas in povidone-iodine group 34 of 40 patients. In the RCT by Delavarian et al., (2019) 50% of grade 3 OM was observed after 3 weeks in the control group whereas only 33% of study group reached this grade after 4 weeks which was statistically significant (P < 0.05). Similarly, Arun et al., (2020) also showed in turmeric treated group none showed grade 3 OM and 73.3% had grade 1 after 4th week of treatment whereas in control group 13% showed grade 3 OM and 68%had grade 2. Adhvaryu et al.,(2018) observed significant decrease in grade III and IV mucositis from 51.6% to 12.8% (P≤ 0.001) among control and curcumin treated group respectively. Shah et al,(2021) showed that both mouthwashes curcumin and Benzydamine were equally effective in preventing the occurrence of severe form of oral mucositis. 

*Grading of Oral Mucositis*

*OMAS*

Patients clinically diagnosed with oral mucositis managed with Curcumin/Turmeric in 3 studies (Patil et al., 2015; Charanthimath, 2016; Saldanha and Almeida 2014) used OMAS for grading.1 study by Saldanha and Almeida (2014) compared score of Treatment Induced Oral Mucositis in pre and post test. The difference between the test were more in turmeric group compared to saline mouth wash proving that turmeric mouth wash was better than saline mouth wash. 1 study by Charanthimath (2016) observed percentage of change in erythema and size of ulcer from minimal changes to total reduction, significant difference was obtained from baseline, first and second week follow ups and another study by Patil et al., (2015) showed erythematous and ulceration scores were statistically significant p=0.050 and p<0.001 from baseline to second follow up.

*WHO Mucositis Score*

WHO Mucositis score were measured in 5 studies (Mansourian et al., 2015; Patil et al., 2015; Charanthimath, 2016; Arun et al., 2020; Adhvaryu et al., 2018; Shah et al., 2021). RCT of Charantimath (2016), observed change in erythema and ulcer healing and showed improvement in first and second week which were statistically significant in curcumin group. A Study by Patil et al., (2015) reported that WHO Scores (p=0.003) were statistically significant from baseline to second follow up.1 study by Adhvaryu et al., (2018) showed a significant reduction in incidence of oral mucositis and in grade III and IV mucositis in curcumin treated group.1 study by Arun et al., (2020) majority of the patients had grade 1 mucositis and none had grade 3 mucositis in turmeric group compared to placebo where majority had grade 2 and few had grade 3 mucositis at the end of fourth week. Study by Shah et al.,2021 majority had developed grade 1 and 2, none had grade 3 mucositis in curcumin group when compared to Benzydamine.

*RTOG Grading*

1 study by Rao et al., (2014) assessed OM using RTOG grading system showed turmeric group had delayed and reduced intolerable mucositis (P<0.001).

*NCI CTC*

The study by Delavarian et al., (2019) had assessed grading of OM by NCI CTC v.2, only 32% of curcumin group developed OM in 2nd week of radiotherapy whereas all in control group. Subjective scale NCI CTCAE were used to assess OM in study by (Arun et el.,2020)

*Pain Assessment*

In two clinical trials comparison between curcumin and chlorhexidine, NRS score showed reduction in pain (p value =0.0001) in a study by Charanthimath (2016) and scores were better from baseline till 2nd follow up(p<0.001) in curcumin group in another study by Patil et al., (2015). VAS analysis showed reduction in burning sensation in topical curcumin group compared to placebo group (Mansourian et al., 2015). 

*Treatment breaks*

Rao et al., (2014) had reported that 17.9% had treatment delay at 6th/7th week of radiation, whereas in Povidone iodine it was 24%.1 study by Adhvaryu et al., (2018) showed that patient completing radiotherapy dose schedule increased in curcumin group of 80%indicating the reduced treatment breaks.

*Treatment days lost*

The study by Rao et al., (2014) observed the days lost during treatment were not statistically significant between povidone iodine and turmeric as it was 7.25 - 0.56 and 7 - 0 days respectively.

*Loss of Body Weight*

The study by Delavarian et al., (2019) showed average loss of body weight as 0.43 ± 0.83 kg in nanomicelle curcumin group compared to control group was 1.32 ± 0.87 (P=0.003). 1 study by Rao et al., (2014) reported weight loss was statistically significant more in Povidone iodine group which was 4.45 ± 2.15, compared to turmeric group which was 3.92 ± 2.13 (P<0.001). 

*Quantitative Synthesis*

**Figure 4:**
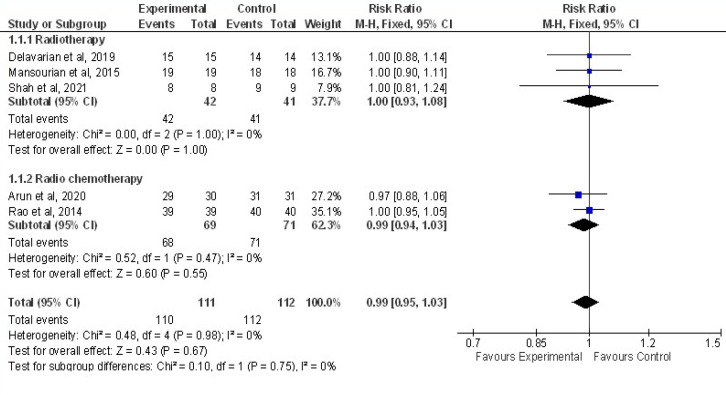
Forest Plot Depicting the Meta-Analysis Results of Curcumin Effect on Overall Incidence of Oral Mucositis Compared with Control in the Prophylactic Phase. Results plotted in left hand side indicate effect in favor of Curcumin and the combined effect including variance is plotted as black diamond at the bottom of forest plot. Analysis was done per subgroup (Radiotherapy and Radiochemotherapy). Events: Patients who had onset of OM

Meta-analysis was performed based on 7 articles with total 283 patients with 141 cases and 142 controls were included. Pooling of five trials were done to assess the effect of curcumin on overall incidence of OM compared to controls. As shown in Figure 4, the random effects model was used and showed that results were not statistically significant (Z=0.43,P=0.67) between curcumin and control group in preventing onset of OM with an overall effect size of 0.99 at 95%CI=0.95,1.03.No heterogeneity of included studies were found. Chi2=0.10,df=1(P=0.75) I^2^=0% Analysis was done per subgroup (Radiotherapy and Radiochemotherapy).Studies done in Radiotherapy (three studies with 83 patients, I^2^=0%) and in Radiochemotherapy (two studies with 140 patients, I^2^=0%. In studies with Radio-chemotherapy with RR of 0.99 at (95%CI=0.94,1.03) and in Radiotherapy with RR of 1.00 at (95%CI=0.93,1.08), irrespective of cancer treatment given curcumin were not effective in preventing overall incidence of OM. 

Pooling of five RCTs were done to assess the effect of curcumin on severity of OM (Grade more or less equal 3) compared to controls is shown in Figure 6. In curcumin group, with 111 patients only 24 had developed severe OM, in control group among 112 patients 95 developed severe OM. Result showed an overall effect size, RR of 0.43(95%CI=0.20,0.91). Result were statistically significant between curcumin and control group Z = 2.20 (P=0.03) in reducing severity of OM . As heterogeneity of included studies were Chi2= 11.16,df=4 (P=0.02) I^2^=64%, subgroup analysis were done with Topical and oral curcumin. Studies done in topical (three studies with 133 patients, I^2^=12%)and in Oral (two studies with 90 patients, I^2^=63%. There was a significant preventive effect of topical curcumin in reducing severity of OM with RR of 0.35 at (95%CI=0.16,0.78) compared to oral with RR of 0.42 at (95%CI=0.04,3.92).

**Figure 5:**
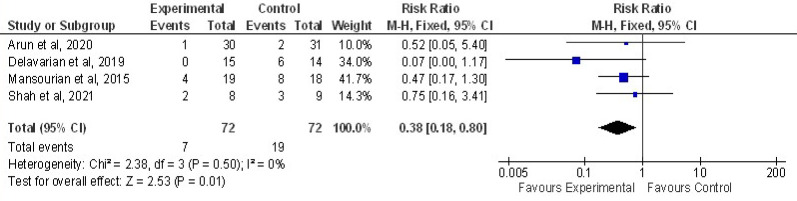
Forest Plot Depicting the Incidence of Delay in Onset of Oral Mucositis (Grade1, week 1) among Curcumin and Control Group

Four Trials were pooled to assess the effect of curcumin on delay in onset of OM compared to controls is shown in Figure 5. Among 72 patients only 7 developed grade 1 OM in curcumin group whereas 19 developed in control group in week 1. Results are statistically significant (Z=2.53, P=0.01) between curcumin and control group in delaying the onset of OM with an overall effect size of 0.38 at 95% CI=0.18,0.80. No heterogeneity of included studies were found. Chi2=2.38, df=3(P=0.50) I^2^=0%

Figure 7 showed pooling of 4 RCTs were done to evaluate the mean difference for OM scores at grade 3 mucositis. OM mean severity reduced with curcumin usage when compared to control, statistically significant (Z=9.55,P<0.00001) with overall effect of -0.85 at 95%CI=-1.02,0.67). No heterogeneity of included studies were observed, Chi2=2.98, df=3(P=0.39), I^2^=0 %. Figure 8 showed combined analysis of two trials of pain intensity from NRS score revealed mean difference of pain reduction with overall effect of -2.17 at 95%CI (-2.77, -1.58) was more in curcumin group when compared to chlorhexidine group statistically significant, Z=7.15(P<0.00001), with heterogeneity Chi2 =1.18, df=1(P=0.28), I^2^ =15% . Combined analysis of two trials of mean change of weight between curcumin and control revealed mean difference with overall effect of -0.78 at (95%CI=-1.30, -0.27) was effective in preventing weight loss in curcumin group when compared to control group statistically significant, Z=2.99(P=0.003), with heterogeneity Chi2 =0.39, df=1(P=0.53), I^2^ =0% as shown in Figure 9.

*Publication Bias*

Figure 10 showed funnel plots indicating publication bias within triangular graph included in the meta-analysis for the comparison of Curcumin and control group. A. indicates no evidence of publication bias among 5 studies identifying overall incidence of OM with subgroup radiotherapy and radiochemotherapy. In B. Delay in onset of OM and C. Mean OM Grading (Grade 3), each included 4 studies showed 1 study is dispersed down might be due to an included smaller trial indicating publication bias.

Adverse effect: From 9 articles only 2 studies reported adverse effect. Mansourian et al., (2015) reported nausea after using the gel, which got resolved after 2 weeks. Adhvaryu et al., (2018) reported mild stomach upset in 10% of cases.

**Table 1:** Characteristics of Included Studies

S No	"Author,Year, Study, Design"	Type of treatment	"PatientDetailsSex/Age"	Sample Size	Curcumin Formulations	Dosage &Directions	Results-Clinical Improvement	Therapy Duration	Adverse Effects	Outcome measures	Grading Scale
1	Suresh Rao et al 2014,Single-blinded, randomized controlled clinical trial	Radiotherapy/Radio-chemotherapy	26-85 years Males-64 Females-16	80 Adults,Group1-40,Group2-40	"Turmeric gargleVsPovidone-iodine"	Turmeric gargle -10 ml,2min,6 times a day.Povidone Iodine-1:100,10ml 2times a day	Decreased intolerable mucositis (P < 0.0001) Lesser incidence of treatment breaks before 4 weeks (P < 0.01) and reduced change in body weight (P < 0.001).	6 weeks	No side-effects	Gargling with turmeric delayed and reduced the severity of mucositis	RTOG
2	Mansourian et al 2015 ,Double blind,placebo-controlled randomized clinical trial	Radiotherapy	Males-31,Females-6	37 Adults	Curcuma longa gel Vs Placebo topical gel	"Gelwas applied for all radiotherapy duration (21 days) 3 times a day,15 min before initiation"	"Erythema-;Case Group-4.9 mm Control Group-8.9 mm;Ulcer-;Case Group-1.3mm; Control group-7.9mm"	8 weeks	Nausea after using gel, got resolved after 2 weeks	"Curcuma Longa improves the grade ofmucositis and reduces the size of oral lesions"	WHO,VAS
3	"Karthikeya Patil et al ,2015; Randomised; Controlled clinical trial"	Radio-chemotherapy	39-70 years,Males-11,Females-9	20 Adults,Group 1-10,Group 2-10	"Chlorhexidine mouthwash VsFreshly prepared curcumin mouthrinse"	Group 1-Chlorhexidine mouthwash 0.2%,1:1,1 min, Thrice daily.Curcumin Mouthrinse 0.0004%,1;5,1min ,thrice daily	Statistically significant difference was found in the NRS (p<0.001), Erythema (p=0.050), ulceration (p<0.001) and WHO scores (p=0.003) between the two groups.	20 days	No side-effects	Curcumin was better than chlorhexidine mouth wash in rapid wound healing, better patient compliance in management of oral mucositis.	"WHO Scale,OMAS,NRS"
4	"Charanthimath ,2016 Randomised; controlled clinical trial; Randomised; controlled clinical trial"	Radio-chemotherapy	_	40 Adults,Group A-20,Group B-20	Curcumin gel Vs Chlorhexidine gel	Group A-Curcumin gel,Group B-Chlorhexidine gel	"Change in ErythemaGroup A- 100% , Group B-57.7%;Size of Ulcer reductionGroup A-103.4%;Group B-53.6% "	2 weeks	No side-effects	Curcumin gel is an effective and safer alternative to chlorhexidine gel in treatment of oral mucositis	"NRS,OMAS,WHO Mucositis scale"
5	"Zahra Delavarian et al,2019Double-blind randomised clinical Trial"	Radiotherapy	Study-62.18+-15.07,Control-55.87+-15.33Females-13; Males-19	"Study Group-16Control Group-16"	"Nanocurcumin Vs Placebo(Lactose)"	1 capsule 80mg/day oral Nanocurcumin(1 capsule of sincurcumin 80mg /day	Statistically significant difference in the severity of mucositis between 2 groups at all visits.	"Day 0,7, 14, 21, 28, 35, 42"	No oral/systemic side effects	Nanomicelle curcumin is an effective agent in prevention of oral mucositis.	NCI-CTC v.2
S No	"Author,Year, Study, Design"	Type of treatment	"PatientDetailsSex/ Age"	Sample Size	Curcumin Formulations	Dosage &Directions	Results-Clinical Improvement	Therapy Duration	Adverse Effects	Outcome measures	Grading Scale
6	"Arun P et al,2020Randomised placebo controlled trial"	Radio-Chemotherapy	"30-90years;Male-28;Female-33;"	"Group A-30Group B -31"	Turmeric extract capsule Vs Placebo	"Group A-500mg turmeric extract thrice dailyGroup B-Placebo"	73.3% had grade 1 mucositis after 4 weeks of treatment. Difference is statistically significant from 3 weeks onwards(p<0.0001)	2 months	No side effects	Turmeric extract reduces the incidence and severity of radiation-induced mucositis.	"WHO,NCI CTCAE"
7	Shah et al 2021, Randomised,Parrallel arm, triple-blinded controlled clinical trial	Radiotherapy	"26-96 yearsMales-54Females-14"	"MIT-Control Group-35Study Group- 33PP-Control Group-9Study Group- 8"	Benzydamine 0.15% mouthwash, Curcumin 0.1% mouthwash,	10ml mouthwash thrice daily for 7 days, freshly prepared for 6 visits.(weekly)	Both mouthwashes were effective in delaying onset and preventing severe RIOM.	6 weeks	Burning sensation in 1 patient	0.1%Curcumin mouthwash significantly delay the onset of RIOM.	WHO
8	"Meghna Adhvaryu et al 2018Case controlled open labelled controlled Trial"	Radiotherapy/Radio-chemotherapy	"10-90 years;Males-174Females-30"	"Group 1-95 ControlGroup 2-109Trial group"	"Conventional Radio-chemotherapy, Oral Curcumin"	Control group -Conventional radio-chemotherapy Trial group -650 mg of oral curcumin fortified with 13mg of piperine /day +conventional therapy for 2 months 3 times/day starting from 3 days before planned radiation.	Significant reduction in incidence of mucositis from 92% to 51% (P ≤ 0.001) Grade III and IV mucositis from 51.6% to 12.8% (P≤ 0.001)	2 months	Mild stomach upset in 10%cases	Curcumin showed a adjuvant protective activity to radio-chemotherapy in HNCP.	WHO
9	"Saldanha et al2014;Quasi experimental pre-post intervention design"	Radiotherapy/Radio-chemotherapy	"31-75 yearsMale-32Female-8"	"40 Adults Group I-20 Group II-20 "	"Turmeric Mouth Wash VsSaline Mouth wash "	"50ml freshly prepared Turmeric mouthwash,Thrice daily for 5 days"	"Reduction in mean scoresPreday=25.4Postday=18.9"	5 days	No-side effects	Turmeric mouth wash was better than saline mouth wash.	"Oral mucositis Assessment checklistPain Scale"

MITو Modified Intention to Treat; PP, Per Protocol Analysis ; Visual Analog Scale(VAS), Numeric Rating Scale(NRS), Radiotherapy Oncology Group (RTOG), World Health Association (WHO) scales, Oral Mucositis Assessment Scale (OMAS), National Cancer Institute Common Toxicity Criteria version 2 scale (NCI-CTC v.2), National Cancer Institute Common Terminology Criteria for Adverse Events, version 4.0(NCI CTCAE assessment V.4, Radiation Induced Oral Mucositis(RIOM),RCT-Radio-chemotherapy,RT-Radiotherapy

**Figure 6:**
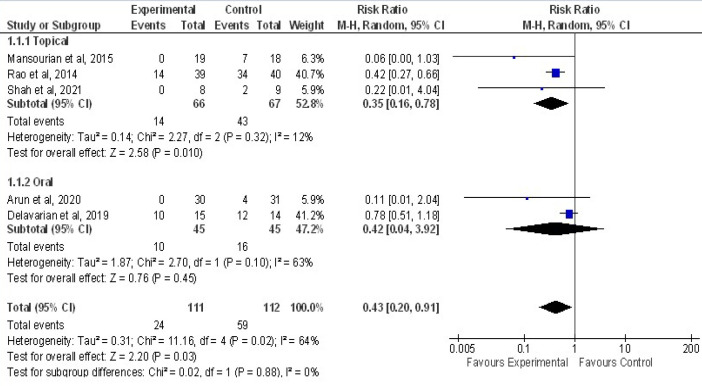
Forest Plot Depicting the Meta-Analysis Results of Curcumin Effect on Incidence of Severe ((Grade>3) Equal or more to 3) Oral Mucositis Compared with Control in Prophylactic Phase. Results plotted in left hand side indicate effect in favor of Curcumin and the combined effect including variance is plotted as black diamond at the bottom of forest plot. Analysis was done per subgroup (Topical and Oral). Events: Patients who had OM severity with grade > 3 more or equal to 3 after treatment

**Figure 7:**
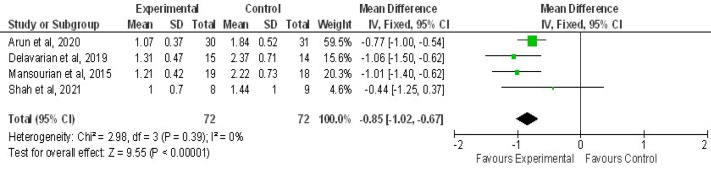
Forest Plot Depicting the Mean Mucositis Grading (Grade 3) among Curcumin and Control group

**Figure 8:**
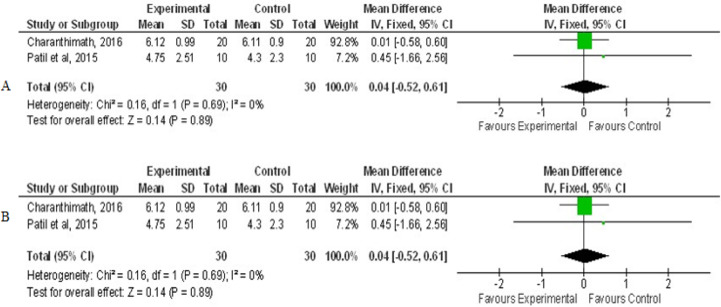
Forest Plot Depicting Pain Scores of Curcumin and Control in Therapeutic Phase. A, Baseline pain scores; B, Second Follow up

**Figure 9:**
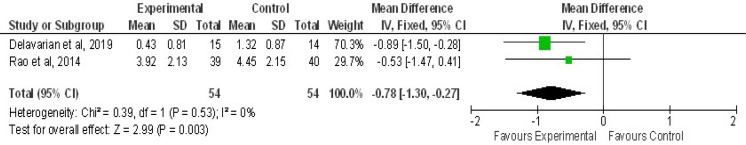
Forest Plot Depicting the Mean Change of Weight loss between Curcumin and Control Group

**Figure 10:**
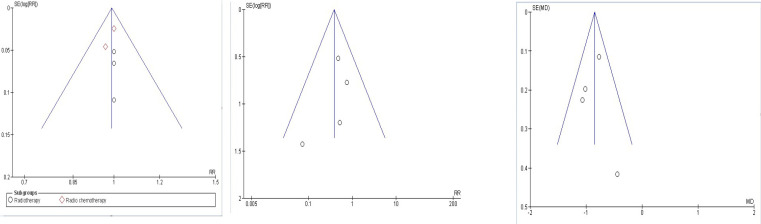
Funnel Plot Depicting Various Studies within Triangular Graph for the Analysis of Publication Bias in the Meta-Analysis for the Comparison of Curcumin and Control Group. A, Overall incidence of OM (5 Studies); B, Delay in onset of OM (4 studies); C. Mean Mucositis Grade-Grade 3 (4 studies)

## Discussion

Most debilitating inevitable dose limiting toxicity of oral cancer therapy such as radiotherapy and chemotherapy is oral mucositis (Rodriguez et al., 2012). Quality of life in these patients are hampered due to its severe complications (Mercadante et al., 2015).

The present meta-analysis included 7 trials finding the efficacy of curcumin in treatment of oral mucositis in patients undergoing radiotherapy, radiochemotherapy for head and neck cancer. The prophylactic use of curcumin effectively delayed the incidence of onset with RR of 0.38 and reduced the severity of oral mucositis with RR of 0.48, statistically significant respectively compared to controls but there was no effectiveness in prevention of the overall incidence of oral mucositis with RR of 0.99, not statistically significant. There was moderate heterogeneity 64% among topical and oral administration due to methodological difference included in formulation and dosages. Oral mucositis mean severity reduced with curcumin usage when compared to control with overall mean difference of-0.85, statistically significant. Prevention of Reduction in weight loss was effective in curcumin over controls with MD of -0.78, statistically significant. 

Cancer treatment induced mucositis cause acute pain due to inflammation, sloughing of tissue and ulcerations. (Lalla et al.,2014). These are subjective measures that has effect in its clinical management. VAS, NRS are reliable and valid pain tool. Therapeutic use of curcumin effectively reduced pain scores graded using NRS in already developed OM with MD of -2.17, statistically significant.

Topical therapies are more easily applied, not expensive and has lesser adverse effects compared to systemic therapies. (Ana et al.,2020). Topical curcuma longa gel prepared by using 500gm of fresh curcumin powder effectively decreased oral symptoms by reducing the size of oral lesions, improving the grades of oral mucositis in HNC patients undergoing radiotherapy (Mansourian et al.,2015). In other study, commercially available Curcumin gel was compared with chlorhexidine gel that had faster healing of wound that was safer and an effective alternative (Charanthimath, 2016). Use of 0.1% of nanocurcumin mouthwash delayed the onset of Oral mucositis (Shah et al.,2021). Curcumin mouthwash 0.004% was better tolerated and effective in reducing the signs and symptoms of chemo-radiotherapy induced oral mucositis (Patil et al.,2015). Turmeric gargle prepared from 400mg turmeric capsule reduced and delayed the severity of OM. They had less treatment breaks and favoured food intake due to reduced mucositis. Frequency of curcumin formulations used thrice to six times showed improvement in oral mucositis and reduced size of oral lesions. (Rao et al.,2014). Saldanha et al.,2014 stated that turmeric mouthwash prepared from 1500mg turmeric powder was better than saline mouthwash. Methodology of formulary preparation for topical turmeric/curcumin among studies varied, hence requires standardised formulation with more RCTs. There was a significant preventive effect of topical curcumin in reducing severity of OM with RR of 0.35 compared to oral with RR of 0.42.

Curcumin has limited bioavailability, poor absorption, metabolised and excreted rapidly when consumed orally. To combat this, Delavarian et al., (2019) in their study considered oral curcumin nanomicelle 80mg/day tablet as an effective and safe agent in preventing the development and reducing the severity of OM, it also has the potency to lower weight loss. Curcumin entrapped nanoparticles produced enhanced oral bioavailability, curcumin solubility in aqueous solutions and absorption achieving the considerable serum and tissue levels and uptake by different tissues (Shaikh et al.,2009). Nanoparticles of curcumin are miscible in water. (Shah et al.,2021). Adhvaryu et al., (2018) suggested oral curcumin to nearly 2000mg/day fortified with piperine enhanced its bioavailability, reducing the incidence of grade III and IV mucositis, reduced drop-out rate with improved patient compliance. Arun et al.,(2020) reported bioavailable turmeric extract 1500mg/day capsule after food reduced the incidence and severity of OM with no systemic toxicity and was safe to use. 

In a recent study by Shah et al., (2021) Modified Intention to treat (MIT) had sample size n=35 in Benzydamine group and n=33 in curcumin group initially, due to large loss to follow up reduced to n=9 in Benzydamine and n=8 in curcumin group. Per protocol(PP) analysis excluded patients who deviated from protocol, but lacked similarity in characteristics can introduce attrition bias. Results may provide low level of evidence due to small sample size but certainly will reflect the reliable estimation of treatment effects. MIT analysis was not taken into consideration as it may overestimate the effect. 

Among six preventive studies (Rao et al.,2014; Mansourian et al., 2015; Delavarian et al., 2019; Arun et al.,2020; Adhvaryu et al., 2018; Shah et al., 2021) using turmeric/curcumin formulations, there were significant benefits than comparator were better in delaying the onset of OM , showed reduction in severity of OM , three (Rao et al., 2014; Delavarian et al., 2019; Adhvaryu et al., 2018) studies found reduction in treatment breaks, weight loss, hence supporting the effectiveness of turmeric/curcumin in treatment of OM. Three studies (Rao et al., 2014; Mansourian et al., 2015; Shah et al., 2021) were topical and three (Delavarian et al., 2019; Arun et al., 2020; Adhvaryu et al., 2018) were oral. Three therapeutic studies (Patil et al., 2015; Charanthimath, 2016; Saldanha and Almeida., 2014) using topical Curcumin/Turmeric showed reduction in Pain, erythema, ulcer size when compared to controls. Two studies (Patil et al., 2015; Charantimath, 2016) with topical curcumin in comparison with chlorhexidine was efficacious, safer and was better in rapid wound healing and better patient compliance. Regular turmeric swish 6 times per day increasing the frequency was effective in preventing oral mucositis (Rao et al., 2014). 9 included studies revealed that turmeric/curcumin as gel, mouthwash, orally was well tolerated, safe and are widely accepted.

From the above 9 clinical trials, Curcumin/Turmeric is effective in controlling signs and symptoms of radio-chemotherapy induced OM in HNC patients with minimal adverse effects. They delay the onset and reduce severity of OM, with less drop-outs, reduced loss of body weight, reduced treatment days. New innovative Curcumin/Turmeric formulations are needed that increase its bioavailability and better effectiveness.

Limitations were though there was difference in cancer treatment, curcumin/turmeric formulations, dosage, concentration, directions, therapy duration, controls, grading scales, follow ups across studies, all 7 trials with only 283 participants, studies published only in English had to be included in this analysis. Due to small sample size and limited randomised controlled trials included, they could have resulted in varying outcome. Overall methodological quality among studies were moderate.

In Conclusion, Curcumin/Turmeric are efficacious, well tolerated and safe in prevention and amelioration of RT/RCT induced oral mucositis in HNC patients. There is moderate to strong evidence that curcumin is effective in delaying the onset and in reducing the severity of OM, mean mucositis severity, pain intensity, weight loss. Dosage required orally was less than 2,000 mg/day of Curcumin/Turmeric, 80mg/day/0.1% mouthwash of nanocurcumin, topically gel/mouthwash used with increase in frequency/day prior and during(prophylactic) and after(therapeutic) RT/RCT are beneficial with no noticeable side effects and are cost effective. However, multi-centred quality randomised controlled trials with innovative curcumin/turmeric formulations are needed to further support the evidence in preventing and treating oral mucositis.
